# *Rhodiola rosea* L. Improves Learning and Memory Function: Preclinical Evidence and Possible Mechanisms

**DOI:** 10.3389/fphar.2018.01415

**Published:** 2018-12-04

**Authors:** Gou-ping Ma, Qun Zheng, Meng-bei Xu, Xiao-li Zhou, Lin Lu, Zuo-xiao Li, Guo-Qing Zheng

**Affiliations:** ^1^Tongde Hospital of Zhejiang province, Hangzhou, China; ^2^Department of Neurology, The Second Affiliated Hospital and Yuying Children's Hospital of Wenzhou Medical University, Wenzhou, China; ^3^School of Chinese Medicine, Hong Kong Baptist University, Hong Kong, China; ^4^Department of Neurology, Affiliated Hospital of Southwest Medical University, Luzhou, China

**Keywords:** *Rhodiola rosea L*., salidroside, learning and memory, cognition, preclinical evidence, possible mechanisms

## Abstract

*Rhodiola rosea* L. (*R. rosea* L.) is widely used to stimulate the nervous system, extenuate anxiety, enhance work performance, relieve fatigue, and prevent high altitude sickness. Previous studies reported that *R. rosea* L. improves learning and memory function in animal models. Here, we conducted a systematic review and meta-analysis for preclinical studies to assess the current evidence for *R. rosea* L. effect on learning and memory function. Ultimately, 36 studies involving 836 animals were identified by searching 6 databases from inception to May 2018. The primary outcome measures included the escape latency in Morris water maze (MWM) test on behalf of learning ability, the frequency and the length of time spent on the target quadrant in MWM test representing memory function, and the number of errors in step down test, dark avoidance test and Y maze test on behalf of memory function. The secondary outcome measures were mechanisms of *R. rosea* L. for learning and/or memory function. Compared with control, the pooled results of 28 studies showed significant effects of *R. rosea* L. for reducing the escape latency (*P* < 0.05); 23 studies for increasing the frequency and the length of time spent on the target quadrant (*P* < 0.05); and 6 studies for decreasing the number of errors (*P* < 0.01). The possible mechanisms of *R. rosea* L. are largely through antioxidant, cholinergic regulation, anti-apoptosis activities, anti-inflammatory, improving coronary blood flow, and cerebral metabolism. In conclusion, the findings suggested that *R. rosea* L. can improve learning and memory function.

## Introduction

Lasting changes in behavior resulting from prior experience can be characterized as the result of learning, memory, and retrieval processes (Thompson, 1986). However, memory is vulnerable across the adult lifespan. A decrease in learning and memory functions is the most common complaint in normal aging process. In a large number of organic diseases, in which there is a physical change in the structure of an organ or part, such as amnesia, Alzheimer's disease (AD) and vascular dementia, the most prominent sign is memory impairment (Thompson, 1986). Currently, there is no valid treatment for cognition impairment in western medicine, although many potential agents exist through novel mechanisms (Parihar and Hemnani, 2004). Cholinesterase inhibitors (ChEIs) and N-methyl-D-aspartate (NMDA) receptor antagonists are first-line pharmacotherapy for mild-to-moderate AD in clinical, with high non-response rate 50–75% (Johnson et al., 2004). Thus, it is urgent to seek new strategies to improve function of memory and cognition.

*Rhodiola rosea* L. (*R. rosea* L.), also known as Rhodiola, Roseroot, Arctic Root, and Golden Root, belongs to the plant family of Crassulaceae, subfamily of sedoideae and genus Rhodiola (Farhath et al., 2005). *R. rosea* L. and its ingredients replenish qi (vital energy), activate blood circulation, unblock blood vessels, enhance mental function, and smooth asthmatic conditions in traditional Chinese medicine (TCM) (Pharmacopoeia Committee of the People's Republic of China Ministry of health, 2005). Salidroside, p-tryosol, rosavin, pyridrde, rhodiosin, and rhodionin are the most unique active ingredients in the Rhodiola species, but vary in the amounts (Zhang et al., 2006). Of the Rhodiola species, *R. rosea* L. has been extensively studied on its phytochemical and toxicological properties (Kurkin and Zapesochnaya, 1985). Modern pharmacological studies indicate that its extracts can increase neurotransmitter level, central nervous system activity, and cardiovascular function. Current studies reported that *R. rosea* L. ingestion can improve cognitive function (Spasov et al., 2000), reduce mental fatigue (Shevtsov et al., 2003), promote free radical mitigation, and exists anti-oxidative (Zhang et al., 2007) and neuroprotective effect (Yu et al., 2008), increase endurance performance (De Bock et al., 2004), and treat symptoms of asthenia subsequent to intense physical and psychological stress (Lazarova et al., 1986). However, the current evidence of *R. rosea* L. for learning and memory function still lack systematic analysis. Thus, we conduct a preclinical systematic review of Rhodiola on learning and memory function to clarify its effectiveness and potential mechanisms on animal models.

## Methods

Preferred Reporting Items for Systematic Review and Meta-Analyses (PRISMA) statement (Stewart et al., 2015) and the Guidelines for reporting systematic reviews and meta-analyses of animal studies (Sena et al., 2014) were abided.

### Database and Literature Search Strategy

Six databases of PubMed, EMBASE, Web of Science, Chinese National Knowledge Infrastructure (CNKI), Wanfangdatabase and VIP information database were electronically searched from the inception up to May 2018. Studies reporting the use of *R. rosea* L. and/or its bioactive ingredients for learning and memory function in animals were identified. Our literature search strategy was as following: 1. Rhodiola (s); 2. *Rhodiola rosea* (s); 3. Roseroot (s); 4. rhodioloside; 5. salidroside; 6. OR/1-5; 7. Memory; 8. Learning; 9. Cognitive function; 10. 6 AND (7 OR 8 OR 9); 11. Animals NOT humans; 12. 10 AND 11.

### Study Selection

Two investigators independently screened the titles and/or abstracts based on the search strategy. Of the search results, we assessed the full-text articles for eligibility. Any uncertainty eligibility was resolved by discussion. Studies were eligible for our systematic review if they met: (1) Animal models were established for learning and memory injury; (2) Analyzed interventions were received *R. rosea* L. and/or its bioactive ingredients as monotherapy at any dose. Comparator interventions were isosteric non-functional liquid (normal saline) or no treatment; (3) the primary measured outcomes were indexes of learning and/or memory function tests, including Morris water maze (MWM), Y maze, step down test, dark avoidance test, active avoidance reaction and one step through test. The secondary outcome measures were mechanisms of *R. rosea* L. for learning and/or memory function. Pre-specified exclusion criteria were as follows: *R. rosea* L. was treated in conjunction with other compounds or *R. rosea* L.-based prescriptions, or without predetermined outcome index, or without *in vivo* model, or without control group, or duplicate publications. In the case of multiple publications from one study, we choose the articles with the largest sample or the earliest publication.

### Data Extraction

The following details were extracted from each included study: (1) the first author's name, publication year; (2) individual data for each study, including animal species, number, sex, and weight; (3) type of animal model and anesthetic used in the model; (4) intervention characteristics, including timing for initial treatment, dosage and method of treatment, duration of treatment, and comparable treatment of control group;(5) main outcome measures on behavior tests and its corresponding *p*-value. For each comparison, we extracted data of mean value and standard deviation from each treatment and control group of every study. If the data for meta-analysis were missing or only expressed graphically, we tried to contact authors for further information or calculated by ourselves if available. Otherwise we only performed qualitative analysis. The data of highest dose was selected when the treatment group included various doses of the target drug. The result of the peak time point was included when the data were expressed at different times.

### Quality Assessment

Two authors independently assessed the methodological quality of the included articles according to the Collaborative Approach to Meta-Analysis and Review of Animal Data from Experimental Studies (CAMARADES) 10-item checklist (Sena et al., 2007): (1) peer-reviewed publication; (2) statements of temperature control; (3) randomization to treatment or control group; (4) blinded induction of model; (5) blinded assessment of outcome; (6) use of anesthetic without significant intrinsic neuroprotective activity; (7) appropriate animal model; (8) sample size calculation; (9) compliance with animal welfare regulations; and (10) declaration of potential conflict of interests. Each study was given an aggregate quality score based on one-point awarding for each item. Discrepancies were resolved by discussion or consultation with corresponding author.

### Statistical Analysis

Meta-analyses and sub-analyses were performed using RevMan 5.3 software. Outcome measures were all considered as continuous data and given an estimate of the combined overall effect sizes utilizing standard mean difference (SMD) with the random effects model. SMD with its 95% confidence interval (CI) was used to assess the strength of efficacy of *R. rosea* L. and/or its bioactive ingredients for learning and memory function. Publication bias was assessed with a funnel plot. To clarify the impact of factors potentially modifying the outcome measures, we also conducted sensitivity analyses and subgroup analyses according to the following variables: animal species, anesthetic used, type of animal model and the treatment time. The I^2^ statistic was used for assessment of heterogeneity among individual studies. Probability value *P* < 0.05 was considered significant.

## Results

### Study Inclusion

**Figure 1:**
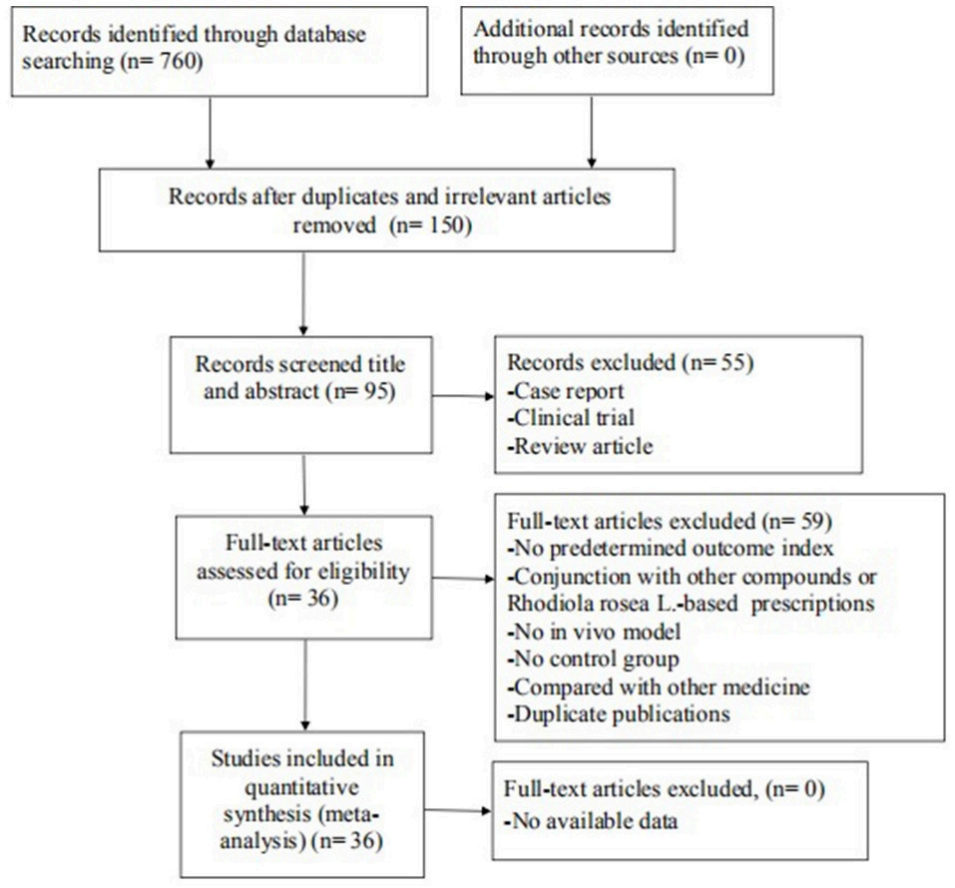
Summary of the process for identifying candidate studies.

We identified 760 potentially relevant articles from the six databases. After removal of duplicates and irrelevant articles, 150 records remained. After going through the titles and abstracts, 55 were excluded because they were case reports, clinical trials or review articles. By reading the remaining full-text articles, 59 articles were excluded if: (1) not predetermined outcome index; (2) not published in peer-review journals; (3) compared with other medicine; (4) no *in vivo* model; (5) no control group; (6) conjunction with other compounds or *R. rosea* L.-based prescriptions. Finally, 36 eligible studies (You et al., 2000; Jiang et al., 2001; Liu et al., 2003, 2017a,b; Xie, 2003; Wu et al., 2004; Deng, 2006; Shi et al., 2006; Chen, 2008; Wang et al., 2008, 2012, 2013; Cao, 2009; Ji et al., 2009; Liu, 2009; Qu et al., 2009; Zou et al., 2009; Mao et al., 2010; Zhao et al., 2010; Yang et al., 2011a,b, 2017; Sun et al., 2012; Zhang S. et al., 2012; Zhang X.X. et al., 2012; Qi et al., 2013; Zhang et al., 2013; Barhwal et al., 2015; Yan et al., 2015; Vasileva et al., 2016; Ge et al., 2017; Guo et al., 2017; Wei, 2017; Xiong and Gao, 2017; Yang, 2017) involving 836 animals were identified (Figure 1).

### Characteristics of Included Studies

The basic characteristics of the included studies are summarized in Table 1. Thirty-six studies included were published between 2000 and 2017 and described comparisons based on three main outcome measures of learning and memory function. For animal species, 27 studies used rats including Sprague-Dawley (SD) rats (Wang et al., 2008; Cao, 2009; Ji et al., 2009; Liu, 2009; Qu et al., 2009; Zou et al., 2009; Yang et al., 2011a,b, 2017; Zhang X.X. et al., 2012; Qi et al., 2013; Zhang et al., 2013; Barhwal et al., 2015; Yan et al., 2015; Liu et al., 2017a,b; Wei, 2017) and Wistar rats (Jiang et al., 2001; Xie, 2003; Chen, 2008; Zhao et al., 2010; Sun et al., 2012; Wang et al., 2012; Vasileva et al., 2016; Ge et al., 2017; Xiong and Gao, 2017; Yang, 2017) as animal models. Eight studies used mice including C57BL/6J (Mao et al., 2010), ICR (Deng, 2006; Zhang X.X. et al., 2012), BALB/C (Liu et al., 2003), Kunming mice (You et al., 2000; Wu et al., 2004; Wang et al., 2013; Ge et al., 2017). The remaining 1 study used mice without mentioning its species (Shi et al., 2006). Seventeen studies (You et al., 2000; Jiang et al., 2001; Liu et al., 2003, 2017a; Xie, 2003; Wu et al., 2004; Deng, 2006; Shi et al., 2006; Wang et al., 2008; Cao, 2009; Ji et al., 2009; Qu et al., 2009; Mao et al., 2010; Sun et al., 2012; Zhang X.X. et al., 2012; Zhang et al., 2013; Yang et al., 2017) induced cognitive impairment by Alzheimer's disease (AD) model, 8 studies (Chen, 2008; Liu, 2009; Zou et al., 2009; Zhang X.X. et al., 2012; Wang et al., 2013; Yan et al., 2015; Liu et al., 2017b; Xiong and Gao, 2017) by vascular dementia (VD) model, 5 studies (Yang et al., 2011b; Qi et al., 2013; Barhwal et al., 2015; Ge et al., 2017; Guo et al., 2017) by hypobaric hypoxia model, 2 studies (Wang et al., 2012; Zhang X.X. et al., 2012) by sleep deprivation model, 2 studies (Zhao et al., 2010; Yang, 2017) by diabetes mellitus (DM) model, 1 study by status epileptics (SE) model (Yang et al., 2011b), 1 study (Wei, 2017) by posttraumatic stress disorder, and the remaining 1 study (Vasileva et al., 2016) by using scopolamine. For anesthesia chosen in experiments, 6 studies (Cao, 2009; Liu, 2009; Zou et al., 2009; Wang et al., 2013; Liu et al., 2017b; Xiong and Gao, 2017) used chloral hydrate, 8 studies (Xie, 2003; Chen, 2008; Qu et al., 2009; Zhang X.X. et al., 2012; Zhang et al., 2013; Wei, 2017; Yang, 2017; Yang et al., 2017) used pentobarbital sodium, 1 study (Yan et al., 2015) used isoflurane,1 study (Wang et al., 2012) used ethyl ether, 3 studies (Liu et al., 2003; Deng, 2006; Zhao et al., 2010) needn't use it because of only neurobehavioral tests being conducted in rats/mice, and the remaining 17 studies did not report it. Thirty-four studies were conducted in China, 1 study (Vasileva et al., 2016) in Bulgaria, and the remaining one (Barhwal et al., 2015) in India. For outcome measures, 28 studies of comparisons reported learning data as escape latency in MWM (Jiang et al., 2001; Liu et al., 2003, 2017a,b; Wu et al., 2004; Deng, 2006; Shi et al., 2006; Chen, 2008; Wang et al., 2008, 2012; Cao, 2009; Ji et al., 2009; Liu, 2009; Qu et al., 2009; Zou et al., 2009; Zhao et al., 2010; Yang et al., 2011a,b, 2017; Sun et al., 2012; Zhang et al., 2013; Barhwal et al., 2015; Yan et al., 2015; Ge et al., 2017; Guo et al., 2017; Wei, 2017; Xiong and Gao, 2017; Yang, 2017), 23 studies of comparisons presented the frequency and/or the length of time spent on the target quadrant in MWM as the indicator of memory ability (Chen, 2008; Wang et al., 2008; Cao, 2009; Ji et al., 2009; Liu, 2009; Qu et al., 2009; Zou et al., 2009; Zhao et al., 2010; Yang et al., 2011a,b, 2017; Sun et al., 2012; Zhang S. et al., 2012; Zhang et al., 2013; Barhwal et al., 2015; Yan et al., 2015; Ge et al., 2017; Guo et al., 2017; Liu et al., 2017a,b; Wei, 2017; Xiong and Gao, 2017; Yang, 2017), and 7 studies (Jiang et al., 2001; Liu et al., 2003; Deng, 2006; Liu, 2009; Zhang X.X. et al., 2012; Wang et al., 2013; Vasileva et al., 2016) of comparisons reported memory outcome measure by the number of errors in step down test, dark avoidance test, the active avoidance test and/or Y maze. Additionally, 3 studies (Wu et al., 2004; Wang et al., 2012; Zhang X.X. et al., 2012) report the reaction time in Y maze. Glutathione (GSH) was reported in 5 studies (Wang et al., 2008; Qu et al., 2009; Yang et al., 2011a,b; Zhang et al., 2013); NADH/NADPH in 2 studies (Zhang et al., 2013; Barhwal et al., 2015); superoxide dismutase (SOD) and/or malondialdehyde (MDA) in 14 studies (Jiang et al., 2001; Shi et al., 2006; Chen, 2008; Qu et al., 2009; Zou et al., 2009; Yang et al., 2011a,b; Zhang S. et al., 2012; Zhang X.X. et al., 2012; Zhang et al., 2013; Liu et al., 2017b; Wei, 2017; Xiong and Gao, 2017; Yang, 2017); NO and/or NOS in 3 studies (Deng, 2006; Chen, 2008; Wang et al., 2013); acetylcholine (Ach) and/oracetylcholinesterase (AchE) in 7 studies (Jiang et al., 2001; Xie, 2003; Wu et al., 2004; Shi et al., 2006; Chen, 2008; Cao, 2009; Zhang et al., 2013); caspase-3 in 3 studies (Qu et al., 2009; Yan et al., 2015; Liu et al., 2017b); tumor necrosis factor-α(TNF-α) in 1 study (Zou et al., 2009); nuclear factor κB (NF-κB) in 1 study (Zhang et al., 2013); Bcl-2 and/or Bax protein in the hippocampus in 5 studies (Cao, 2009; Yan et al., 2015; Guo et al., 2017; Liu et al., 2017a; Wei, 2017).

**Table 1:** Characteristics of included 36 studies.

**Study**	**Species (Sex, *n* = trial/control group)**	**Weight**	**Modeling approach**	**Anesthetic**	**Intervention**		**Outcome measure**	**Intergroup Differences**
					**Trial group**	**Control group**		
1. You et al., 2000	Kunming mice (male,10/10)	25 ± 2 g	Cognitive impairment induced by i.p. SCOP (1 mg/kg); by i.g. 30% ethanol (0.1 ml/10 g); by s.i. sodium nitrite (120 mg/kg)	NR	*R. rosea* L., i.g. 200 mg/kg/day for 30 days before the model	Normal saline	1. Error latency in SDT 2. Error latency in DAT	1. *P* < 0.01 2. *P* < 0.01
2. Jiang et al., 2001	Wistar rats (male, 8/8)	445.35 ± 625.73 g	Cognitive Impairment induced by i.p SCOP (2 mg/kg)	NR	*R. rosea* L., i.m. 15 mg/kg/day for 4 weeks before the model	Normal saline	1. Escape latency in MWM 2. The number of errors in SDT 3. Ach, ChAT 4. LPO, SOD	1. *P* < 0.05 2. *P* < 0.05 3. *P* < 0.05 4. *P* < 0.05
3. Liu et al., 2003	BALB/c mice (male, 10/10)	20–25 g	Cognitive impairment induced by i.p SCOP;	No need	Rhodiola henryi Extract, i.g. 0.1, 0.3, 0.5 g/kg/day for 30 days before the model	Distilled water	1. Escape latency in MWM 2. The number of errors in DAT	1. *P* < 0.05 2. *P* < 0.01
	BALB/c mice (male, 10/10)	20–25 g	Pre-treatment with normal mice	No need	Rhodiola henryi Extract, i.g. 0.1, 0.3, 0.5 g/kg/day for 30 days	Distilled water	1. Escape latency in MWM 2. The number of errors in DAT 3. The number of errors in SDT	1. *P* < 0.05 2. *P* > 0.05 3. *P* > 0.05
4. Xie, 2003	Wistar rats (male, 10/10)	131.7 ± 12.2 g	AD model induced by bilateral hippocampal injection Aβ _1−40_ and i.p. D-gal	2.5% pentobarbital sodium (40 nmg/kg)	*R. rosea* L., i.p. 15 mg/kg/day for 4 weeks accompanying the model	Normal saline	1. Reaction time in Y maze 2. Escape latency in one step through test 3. AchE	1. *P* < 0.05 2. *P* < 0.05 3. *P* < 0.01
5. Wu et al., 2004	Kunming mice (male and female, 12/12)	18–20 g	Cognitive impairment induced by i.p SCOP (2 mg/kg)	NR	R. rosea L. extract, i.g. 1.27, 3.81, 11.41 g/kg/day for 2 weeks before the model	CMC-Na	1. Escape latency in MWM 2. AchE	1. *P* < 0.05 2. *P* < 0.01
6. Shi et al., 2006	mice (male, 10/10)	NR	Cognitive impairment induced by i.p SCOP (5 mg/kg)	NR	R. rosea L. extract, i.g. 3.81 g/kg/day for 3 weeks before the model	CMC-Na	1. Escape latency in MWM 2. AchE 3. SOD, MDA, MAO	1. *P* < 0.01 2. *P* < 0.01 3. *P* < 0.01
7. Deng, 2006	ICR mice (male and female, 27/28)	20 ± 2 g	Cognitive impairment induced by i.p SCOP (2 mg/kg)	No need	*R. rosea* L., i.g. 0.1, 0.6 g/kg/day for 15 days before the model	Distilled water	1. Escape latency in MWM 2. The number of errors in SDT	1. *P* < 0.01 2. *P* < 0.01
8. Chen, 2008	Wistar rats (male, 8/8)	250 g	Bilateral permanent occlusion of the common carotid arteries	0.4% pentobarbital sodium (1 ml/100 g)	*R. rosea* L., i.g. 5 g/kg/day for 28 days after the model	Distilled water	1. Escape latency in MWM 2. The number of target platform crossings 3. Time spent in target quadrant 4. SOD, MDA 5. AchE 6. Neuronal apoptosis	1. *P* < 0.05 2. *P* < 0.05 3. *P* < 0.05 4. *P* < 0.05 5. *P* < 0.05 6. *P* < 0.05
9. Wang et al., 2008	SD rats (male, 12/11)	250–300 g	AD model induced by D-gal +AlCl_3_+SCOP	NR	*R. rosea* L., i.g. 5 g/kg/day for 4 weeks after the model	Normal saline	1. Escape latency in MWM 2. The number of target platform crossings 3. Time spent in target quadrant 4. CAT, GSH-Px	1. *P* < 0.05 2. *P* < 0.05 3. *P* < 0.05 4. *P* < 0.05
10. Cao, 2009	SD rats (male, 12/12)	250 ± 20 g	AD model induced by D-gal +AlCl_3_ +SCOP	10% chloral hydrate (3.5 ml/kg)	*R. rosea* L., i.g. 5 g/kg/day for 28 days after the model	Normal saline	1. Escape latency in MWM 2. The number of target platform crossings 3. Time spent in target quadrant 4. AchE 5. NOS 6. Bax, Bcl-2	1. *P* < 0.01 2. *P* < 0.01 3. *P* < 0.01 4. *P* < 0.01 5. *P* < 0.01 6. *P* < 0.01
11. Ji et al., 2009	SD rats (male,12/11)	250–300 g	AD model induced by D-gal +AlCl_3_ +SCOP	NR	*R. rosea* L., i.g. 10 g/kg/day for 4 weeks after the model	Distilled water	1. Escape latency in MWM 2. The number of target platform crossings 3. Time spent in target quadrant	1. *P* < 0.05 2. *P* < 0.05 3. *P* < 0.05
12. Liu, 2009	SD rats (male, 11/11)	240–300 g	Cerebralhypoperfusion by MCAO for 3 h	4% chloral hydrate (1 ml/100 g)	R. rosea L.,i.p. 12 mg/day for 10 days before the model	Normal saline	1. Escape latency in MWM 2. Time spent in target quadrant 3. Ach	1. *P* > 0.05 2. *P* > 0.05 3. *P* < 0.01
13. Qu et al., 2009	SD rats (male, 12/12)	240–260 g	AD model induced by bilateral ICV with STZ (1.5 mg/kg)	1% pentobarbital sodium (40 mg/kg)	R. rosea L. crenulate extracts, i.g. 1.5, 3.0, 6.0 mg/kg, twice a day for 21 days before the model	CMC-Na	1. Escape latency in MWM 2. Time spent in target quadrant 3. GSH, GR, MDA 4. ATP, COX 5. Neuronal apoptosis 6. Caspase-3, NeuN	1. *P* < 0.05 2. *P* < 0.05 3. *P* < 0.05 4. *P* < 0.05 5. *P* < 0.05 6. *P* < 0.05
14. Zou et al., 2009	SD rats (male, 15/15)	300 ± 20 g	VD model induced by bilateral CCAO for 10 min	10% chloral hydrate (400 mg/kg)	R. rosea L., i.p. 12 mg/kg/day for 7 days before surgery	Normal saline	1. Escape latency in MWM 2. Time spent in target quadrant 3. SOD, MDA 4. TNF-α	1. *P* < 0.05 2. *P* < 0.05 3. *P* < 0.05 4. *P* < 0.05
15. Mao et al., 2010	C57BL/6J mice (female, 10/10)	5-month-old mice	Aging model induced by s.i. D-gal (50 mg/kg)	NR	*R. rosea* L., i.g. 1 g /kg/day for 8 weeks accompanying the model	PBS	1. The number of errors in SDT 2. GFAP, NT-3 3. Splenic T Lymphocyte 4. Proliferation and IL-2 Activity	1. *P* > 0.05 2. *P* < 0.01 3. *P* < 0.01
16. Zhao et al., 2010	Wistar rats (male, 10/10)	200–250 g	DM model induced by i.p. STZ	No need	R. rosea L., i.g., 50 mg/kg for 12 weeks after the model	Normal saline	1. Escape latency in MWM 2. The number of target platform crossings 3. Time spent in target quadrant	1. *P* < 0.01 2. *P* > 0.05 3. *P* < 0.05
17. Yang et al., 2011b	SD rats (male, 8/8)	190–250 g	Status epilepticus model induced by i.p. lithium chloride + pilocarpine	NR	*R. rosea* L., i.p. 1 g/kg/day for 7 days (1 day before the model)	Normal saline	1. Escape latency in MWM 2. Time spent in target quadrant 3. SOD, MDA, GSH,GSH-Px	1. *P* < 0.05 2. *P* < 0.05 3. *P* < 0.05
18. Yang et al., 2011a	SD rats (male, 10/10	180–220 g	Hypobaric hypoxia	NR	*R. rosea* L., i.p. 1 g/kg/day for 34 days accompanying the model	Normal saline	1. Escape latency in MWM 2. Time spent in target quadrant 3. SOD, MDA, GSH, GSH-Px	1. *P* < 0.05 2. *P* < 0.05 3. *P* < 0.05
19. Sun et al., 2012	Wistar rats (male, 9/8)	350 ± 20 g	AD model induced by D-gal+AlCl_3_+SCOP	NR	*R. rosea* L., i.g. 5 g/kg/day for 4 weeks after the model	Distilled water	1. Escape latency in MWM 2. The number of target platform crossings 3. Time spent in target quadrant	1. *P* < 0.05 2. *P* < 0.05 3. *P* < 0.05
20. Wang et al., 2012	Wistar rats (male, 5/5)	190–230 g	Sleep deprivation induced by MMPM	Ethyl ether	*R. rosea* L., i.g. 180 mg/kg/day until sacrifice (10 days before the model)	Normal saline	1. Reaction time in Y maze	2. *P* < 0.05
21. Zhang S. et al., 2012	ICR mice (male and female, 10/10)	21.4 ± 2 g	Cognitive impairment induced by i.p. SCOP (1 mg/kg); by i.g. 40% ethanol (0.2 ml)	NR	*R. rosea* L. compound i.g. 1.2 g/kg/day for 28 days before the model	Normal saline	1. Time spent in target quadrant of MWM 2. SOD, NO	1. *P* < 0.05 2. *P* < 0.05
22. Zhang X.X. et al., 2012	SD rats (male, 6/6)	240–270 g	Sleep deprivation induced by MMPM	0.4% pentobarbital sodium (40 mg/kg)	*R. rosea* L., i.p. 10 ml/kg/day for 3 days before the model	Normal saline	1. Reaction time in Y maze 2. The number of errors 3. SOD, MDA 4. Neuronal apoptosis 5. AchE	1. *P* < 0.05 2. *P* < 0.05 3. *P* < 0.05 4. *P* < 0.05 5. *P* < 0.05
23. Zhang et al., 2013	SD rats (male, 8/8)	300 ± 15 g	AD model induced by bilateral hippocampal injection A β _1−40_ with 10 ug	1% pentobarbital sodium (40 mg/kg)	*R. rosea* L., i.p. 25, 50, 75 mg/kg/day for 21 days after the model	Normal saline	1. Escape latency in MWM 2. The number of target platform crossings 3. Time spent in target quadrant 4. SOD, MDA, GSH-Px 5. Ach, AchE 6. NADH/NADPH 7. nuclear factor κB	1. *P* < 0.01 2. *P* < 0.01 3. *P* < 0.01 4. *P* < 0.01 5. *P* < 0.01 6. *P* < 0.01 7. *P* < 0.01
24. Qi et al., 2013	SD rats (male, 10/10)	180–200 g	Hypobaric hypoxia	NR	*R. rosea* L., i.g. 1 g/100 g, twice a day for 2 weeks before the model	Normal saline	1. AAR retention 2. Neuronal apoptosis	1. *P* < 0.05 2. *P* < 0.05
25. Wang et al., 2013	Kunming mice (male and female, 10/10)	18–22 g	VD model by bilateral CCAO for 20 min*2	4%chloralhydrate(400 mg/kg)	*R. rosea* L., i.g. 60 mg/kg/day for 25 days after the model	Distilled water	1. Escape latency in MWM 2. The number of errors in SDT 3. NOS, NO	1. *P* < 0.01 2. *P* < 0.01 3. *P* < 0.01
26. Yan et al., 2015	SD rats (male, 12/12)	240 ± 20 g	VD model by bilateral permanent CCAO	isoflurane	*R. rosea* L., i.p. 20 mg/kg/day for 35 days (1 day before the model)	Normal saline	1. Escape latency in MWM 2. Time spent in target quadrant 3. Caspase-3 4. Bax/Bcl-2	1. *P* < 0.05 2. *P* < 0.05 3. *P* < 0.05 4. *P* < 0.05
27. Barhwal et al., 2015	SD rats (male, 12/12)	220 ± 10 g	Hypobaric hypoxia	NR	*R. rosea* L., i.g. 25 mg/kg/day for 22 days (8 days before the model)	Normal saline	1. Escape latency in MWM 2. The number of target platform crossings 3. Time spent in target quadrant 4. NADH/NADPH	1. *P* < 0.01 2. *P* < 0.01 3. *P* < 0.01 4. *P* < 0.01
28. Vasileva et al., 2016	Wistar rats (male, 10/10)	160–200 g	Scopolamine-impaired memory model	No need	*R. rosea* L., i.g., 50, 100 mg/kg for 12 days after the model	Normal saline	1. Escape times in AAR 2. Number of intertrial crossings in AAT	1. *P* > 0.05 2. *P* > 0.05
29. Ge et al., 2017	Wistar rats (male, 9/9)	NR	Hypobaric hypoxia	No need	*R. rosea* L., i.g., 40 mg/kg for 28 days after the model	Normal saline	1. Escape latency in MWM 2. The number of target platform crossings	1. *P* < 0.05 2. *P* < 0.05
30. Liu et al., 2017a	SD rats (male, 10/10)	260 ± 20 g	AD model induced by i.h. NaN_3_	NR	*R. rosea* L., i.g., 15 mg/kg for 28 days after the model	Normal saline	1. Escape latency in MWM 2. Time spent in target quadrant 3. AKT, GSK-3β 4. (p-AKT, p-GSK-3β) 5. Bax, Bcl-2	1. *P* < 0.05 2. *P* < 0.05 3. *P* < 0.05 4. *P* < 0.05
31. Liu et al., 2017b	SD rats (male, 10/10)	260 ± 20 g	VD model induced by CCAO	10% chloral hydrate	*R. rosea* L., i.g., 15 mg/kg for 28 days after the model	Normal saline	1. Escape latency in MWM 2. Time spent in target quadrant 3. SOD, MDA 4. p38 5. Caspase-3	1. *P* < 0.05 2. *P* < 0.05 3. *P* < 0.05 4. *P* < 0.05 5. *P* < 0.05
32. Wei, 2017	SD rats (male, 10/10)	230 ± 25 g	PTSD model induced by single prolonged stress	1% pentobarbital sodium	*R. rosea* L., i.g., 25, 50, 75 mg/kg for 14 days after the model	Normal saline	1. Escape latency in MWM 2. The number of target platform crossings 3. Neuronal apoptosis 4. SOD, MDA 5. Bax, Bcl-2, Synapsin I, p-CREB	1. *P* < 0.01 2. *P* < 0.05 3. *P* < 0.05 4. *P* < 0.05 5. *P* < 0.05
33. Yang et al., 2017	SD rats (male, 16/16)	250 ± 24 g	AD model induced by bilateral hippocampal injection Aβ _1−40_	1% pentobarbital sodium (40 mg/kg i.p.)	*R. rosea* L., i.g., 25, 50, 100 mg/kg for 21 days after the model	Normal saline	1. Escape latency in MWM 2. The number of target platform crossings 3. Aβ 4. p75NTR, p-JNK	1. *P* < 0.01 2. *P* < 0.01 3. *P* < 0.01 4. *P* < 0.01
34. Guo et al., 2017	Kunming mice (male/female, 30/30)	21.4 ± 2.2 g	Hypobaric hypoxia	No need	*R. rosea* L., i.g., 200 mg/kg for 56 days after the model	Normal saline	1. Escape latency in MWM 2. Time spent in target quadrant 3. Bax, Bcl-2	1. *P* < 0.05 2. *P* < 0.05 3. *P* < 0.05
35. Yang, 2017	Wistar rats (male, 10/10)	200–250 g	DM model induced by i.p. STZ	1% pentobarbital sodium	*R. rosea* L., i.g., 50 mg/kg for 84 days after the model	Normal saline	1. Escape latency in MWM 2. The number of target platform crossings 3. SOD, MDA	1. *P* < 0.05 2. *P* < 0.05 3. *P* < 0.05
36. Xiong and Gao, 2017	Wistar rats (male,15/15)	257 ± 29 g	VD model induced by CCAO	10% chloral hydrate	*R. rosea* L., i.g., 10 mg/kg for 28 days after the model	Normal saline	1. Escape latency in MWM 2. The number of target platform crossings 3. Time spent in target quadrant 4. SOD, MDA, MAO 5. COX-2, NF- κB	1. *P* < 0.05 2. *P* < 0.05 3. *P* > 0.05 4. *P* < 0.05 5. *P* < 0.05

*AAR, active avoidance reaction; Ach, acetylcholine; AchE, Acetyl cholinesterase;AD, Alzheimer's disease; AlCl3, aluminum trichloride; CCAO, common carotid artery occlusion; ChAT, acetylcholine transferase; CMC-Na, sodium carboxymethylcellulose; DAT, dark avoidance test; D-gal, D-galactose; DM:Diabetes mellitus; ICV, intracerebroventricular injection; i.g., intra-gastrical injection; i.m.:intramuscular injection; i.p., intra-peritoneal injection; i.h., hypodermic injection; LPO, lipid peroxide; MWM, Morris water maze; MCAO, middle cerebral artery occlusion; MDA, Malondialdehyde; MMPM, modified multiple platform method; NR, not report; SCOP:scopolamine; MCAO:middle cerebral artery occlusion; PBS, phosphate buffer saline; PTSD, posttraumatic stress disorder; SDT, step down test; s.i., subcutaneous injection; STZ, streptozotocin; SOD, superoxide dismute; VD, vascular dementia*.

### Study Quality

The score of study quality checklist items ranged from 1/10 to 6/10 in Table 2. Of which, 1 study (Vasileva et al., 2016) obtained 6 points, 10 studies obtained 5 points (Chen, 2008; Zou et al., 2009; Wang et al., 2012, 2013; Zhang X.X. et al., 2012; Zhang et al., 2013; Barhwal et al., 2015; Yan et al., 2015; Xiong and Gao, 2017; Yang et al., 2017), 9 studies (Liu et al., 2003, 2017b; Deng, 2006; Cao, 2009; Liu, 2009; Qu et al., 2009; Mao et al., 2010; Zhao et al., 2010; Guo et al., 2017) obtained 4 points, 8 studies (Jiang et al., 2001; Xie, 2003; Wu et al., 2004; Ji et al., 2009; Ge et al., 2017; Liu et al., 2017a; Wei, 2017; Yang, 2017) obtained 3 points, 7 studies (Shi et al., 2006; Wang et al., 2008; Yang et al., 2011a,b; Sun et al., 2012; Zhang S. et al., 2012; Qi et al., 2013) obtained 2 points, and the remaining one (You et al., 2000) obtained 1 point. Seven studies (Xie, 2003; Deng, 2006; Chen, 2008; Cao, 2009; Liu, 2009; Qu et al., 2009; Wei, 2017) are master's or doctoral thesis, and remaining studies were published in peer-reviewed journals or databases. Twenty-one studies (Liu et al., 2003, 2017a,b; Xie, 2003; Deng, 2006; Chen, 2008; Cao, 2009; Ji et al., 2009; Liu, 2009; Qu et al., 2009; Zou et al., 2009; Wang et al., 2012, 2013; Zhang X.X. et al., 2012; Zhang et al., 2013; Barhwal et al., 2015; Yan et al., 2015; Vasileva et al., 2016; Wei, 2017; Xiong and Gao, 2017; Yang et al., 2017) described control of the room temperature. Except six studies (You et al., 2000; Yan et al., 2015; Liu et al., 2017a,b; Xiong and Gao, 2017; Yang, 2017), the remaining studies declared that they had random allocation to treatment and control groups. Twenty-one studies (Liu et al., 2003, 2017b; Xie, 2003; Deng, 2006; Chen, 2008; Cao, 2009; Liu, 2009; Qu et al., 2009; Zou et al., 2009; Zhao et al., 2010; Wang et al., 2012, 2013; Zhang X.X. et al., 2012; Zhang et al., 2013; Yan et al., 2015; Vasileva et al., 2016; Guo et al., 2017; Wei, 2017; Xiong and Gao, 2017; Yang, 2017; Yang et al., 2017) used anesthetic without significant intrinsic vascular protection activity. Animal model with aged rats/ mice was used in 2 studies (Jiang et al., 2001; Mao et al., 2010), with DM rats in 2 studies (Zhao et al., 2010; Yang, 2017). Thirteen studies (Wu et al., 2004; Chen, 2008; Zou et al., 2009; Mao et al., 2010; Wang et al., 2012, 2013; Zhang X.X. et al., 2012; Zhang et al., 2013; Barhwal et al., 2015; Yan et al., 2015; Vasileva et al., 2016; Xiong and Gao, 2017; Yang et al., 2017) mentioned compliance with animal welfare regulations. Thirteen studies (Deng, 2006; Chen, 2008; Cao, 2009; Liu, 2009; Qu et al., 2009; Barhwal et al., 2015; Yan et al., 2015; Vasileva et al., 2016; Ge et al., 2017; Guo et al., 2017; Liu et al., 2017a,b; Xiong and Gao, 2017) contained statements on potential conflict of interests. There was neither study reporting that if the model establishment and outcome assessment were conducted in double-blind trial or not, nor calculating sample size in the animal experiment.

**Table 2:** Risk of bias of the included studies.

**Study**	**A**	**B**	**C**	**D**	**E**	**F**	**G**	**H**	**I**	**J**	**Total**
1. You et al., 2000	√										1
2. Jiang et al., 2001	√		√				√				3
3. Liu et al., 2003	√	√	√			√					4
4. Xie, 2003		√	√			√					3
5. Wu et al., 2004	√		√						√		3
6. Shi et al., 2006	√		√								2
7. Deng, 2006		√	√			√				√	4
8. Chen, 2008		√	√			√			√	√	5
9. Wang et al., 2008	√		√								2
10. Cao, 2009		√	√			√				√	4
11. Ji et al., 2009	√	√	√								3
12. Liu, 2009		√	√			√				√	4
13. Qu et al., 2009		√	√			√				√	4
14. Zou et al., 2009	√	√	√			√			√		5
15. Mao et al., 2010	√		√				√		√		4
16. Zhao et al., 2010	√		√			√	√				4
17. Yang et al., 2011b	√		√								2
18. Yang et al., 2011a	√		√								2
19. Sun et al., 2012	√		√								2
20. Wang et al., 2012	√	√	√			√			√		5
21. Zhang S. et al., 2012	√		√								2
22. Zhang X.X. et al., 2012	√	√	√			√			√		5
23. Zhang et al., 2013	√	√	√			√			√		5
24. Qi et al., 2013	√		√								2
25. Wang et al., 2013	√	√	√			√			√		5
26. Yan et al., 2015	√	√				√			√	√	5
27. Barhwal et al., 2015	√	√	√						√	√	5
28. Vasileva et al., 2016	√	√	√			√			√	√	6
29. Ge et al., 2017	√		√							√	3
30. Liu et al., 2017a	√	√								√	3
31. Liu et al., 2017b	√	√				√				√	4
32. Wei, 2017		√	√			√					3
33. Yang et al., 2017	√	√	√			√			√		5
34. Guo et al., 2017	√		√			√				√	4
35. Yang, 2017	√					√	√				3
36. Xiong and Gao, 2017	√	√				√			√	√	5

*Studies fulfilling the criteria of: A, peer reviewed publication; B, control of temperature; C, random allocation to treatment or control; D, blinded induction of model; E, blinded assessment of outcome; F, use of anesthetic without significant intrinsic neuroprotective activity; G, animal model (aged, diabetic, or hypertensive); H, sample size calculation; I, compliance with animal welfare regulations; J, statement of potential conflict of interests*.

### Effectiveness

**Figure 2:**
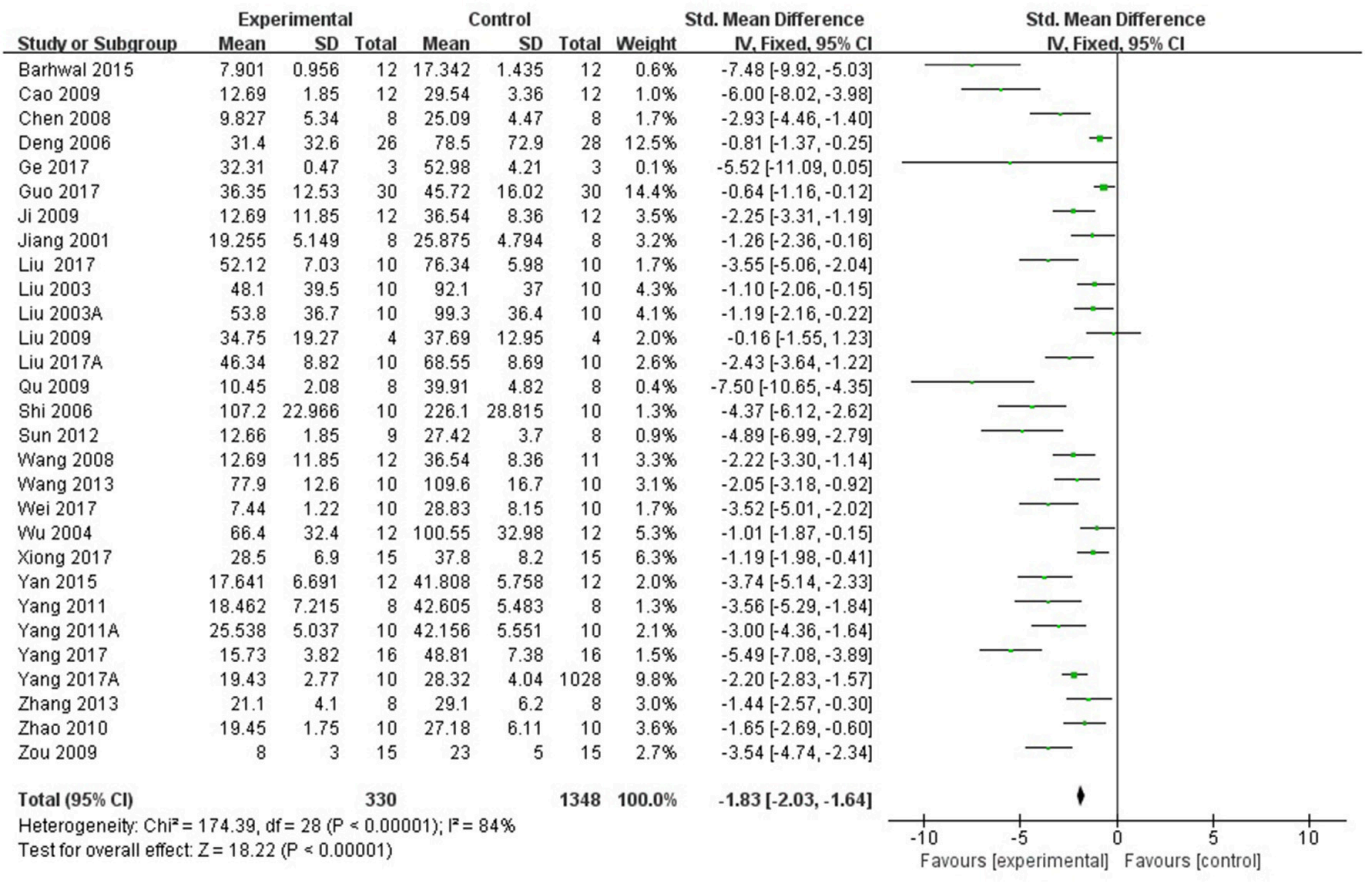
The forest plot: effects of *Rhodiola rosea* L. for decreasing the escape latency in MWM compared with control group.

**Figure 3:**
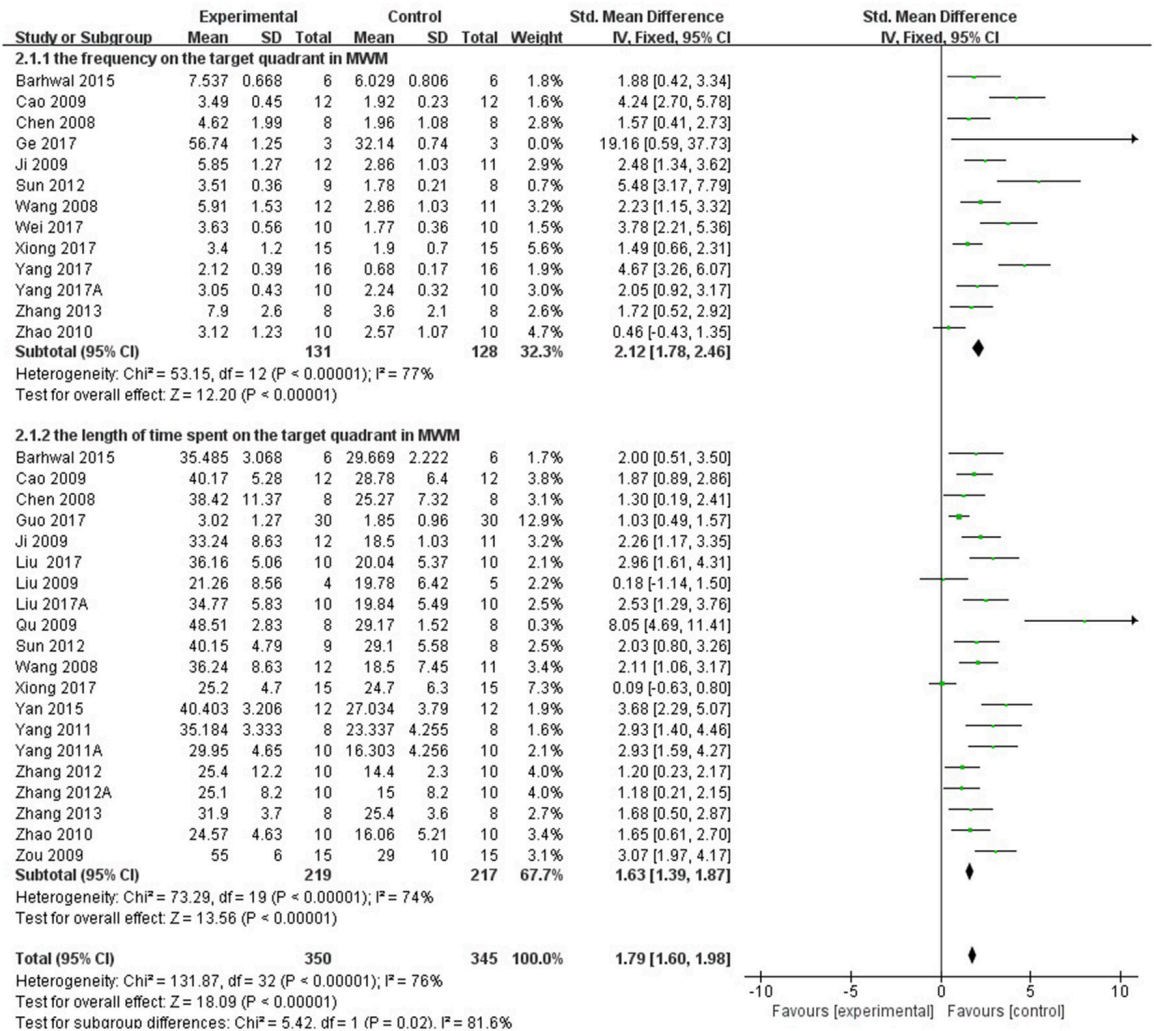
The forest plot: effects of *Rhodiola rosea* L. for decreasing the frequency and the length of time spent on the target quadrant in MWM compared with control group.

**Figure 4:**
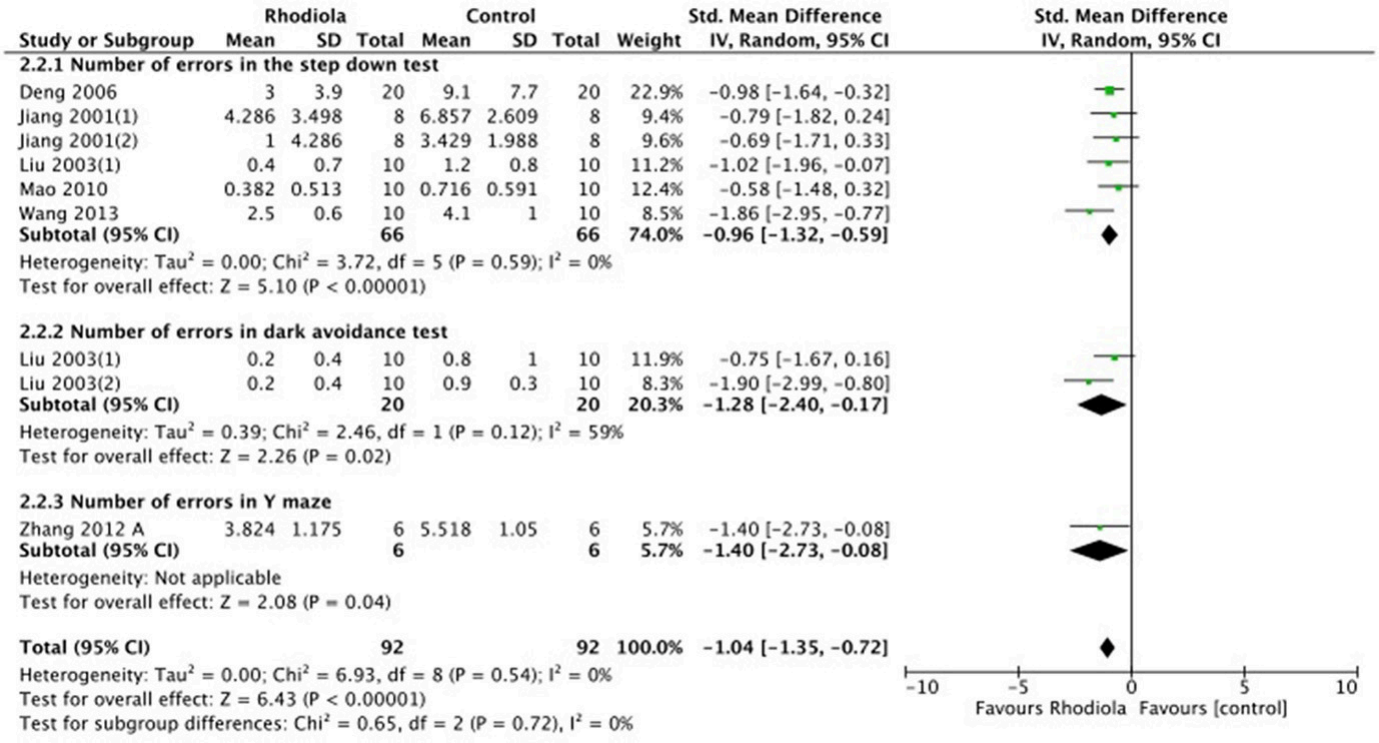
The forest plot: effects of *Rhodiola rosea* L. For decreasing the number of errors compared with control group.

Twenty-eight studies reported the escape latency in MWM as the outcome measure of learning ability included in the analysis. We pooled the whole data to process and found a significant difference in favor of *R. rosea* L. treatment compared with control groups (*P* < 0.00001; SMD = −1.83, 95% CI [−2.03, −1.64]; Heterogeneity: χ^2^ = 174.39, df = 28 (*P* < 0.00001); *I*^2^ = 84%, Figure 2). Twenty-three studies reported the frequency and/or the length of time spent on the target quadrant as the indicator of memory ability. The pooled result showed that *R. rosea* L. significantly increased the frequency and the length of time spent on the target quadrant in MWM (*P* < 0.00001; SMD = 1.79, 95% CI [1.60, 1.98]; Heterogeneity: χ^2^ = 131.87, df = 32 (*P* < 0.00001); *I*^2^ = 76%, Figure 3). Seven studies reported memory outcome measure by the number of errors in step down test, dark avoidance test, the active avoidance test and Y maze. The pooled data showed that *R. rosea* L. resulted in a significant depression on the number of errors when comparing to that in control groups (*P* < 0.00001; SMD = −1.04, 95% CI [−1.35, −0.72]; Heterogeneity: χ^2^ = 6.93, df = 8 (*P* = 0.54); *I*^2^ = 0%, Figure 4).

### Mechanisms of *Rhodiola rosea* for Learning and Memory Function

**Figure 5:**
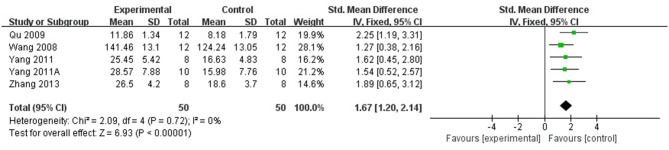
The forest plot: effects of *Rhodiola rosea* L. for increasing glutathione compared with control group.

**Figure 6:**
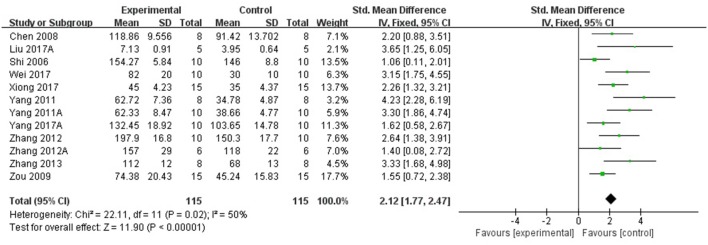
The forest plot: effects of *Rhodiola rosea* L. for increasing superoxide dismutase compared with control group.

**Figure 7:**
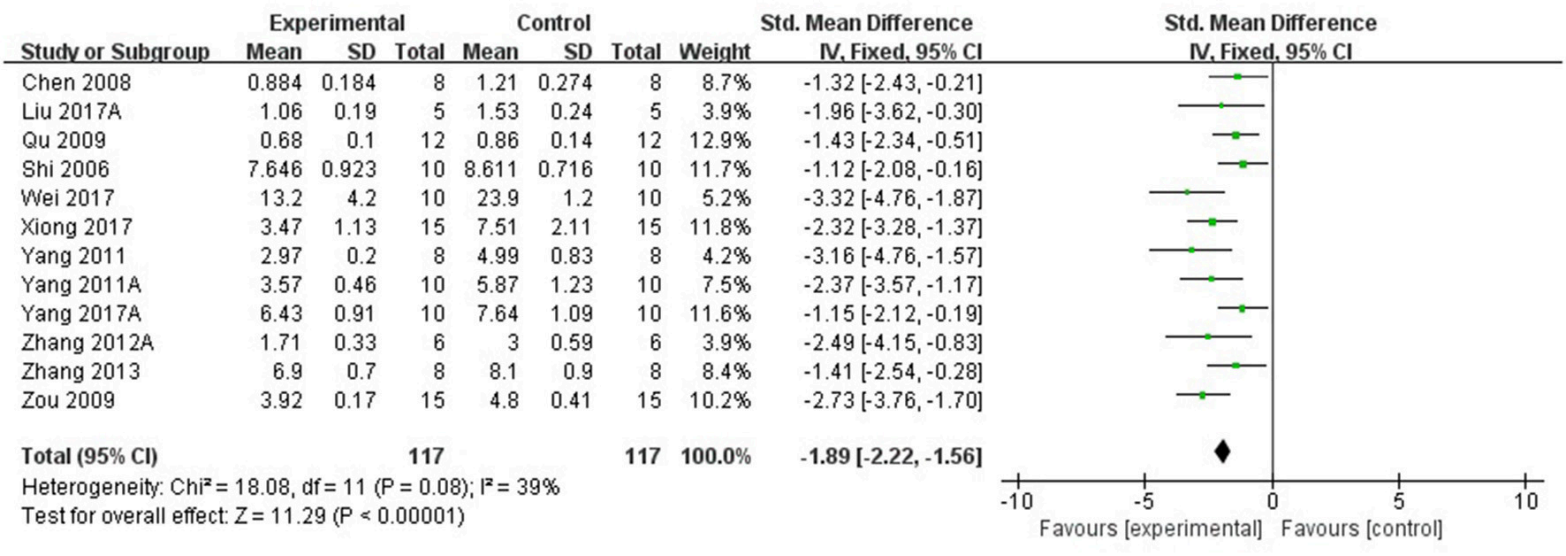
The forest plot: effects of *Rhodiola rosea* L. for decreasing malondialdehyde compared with control group.

**Figure 8:**
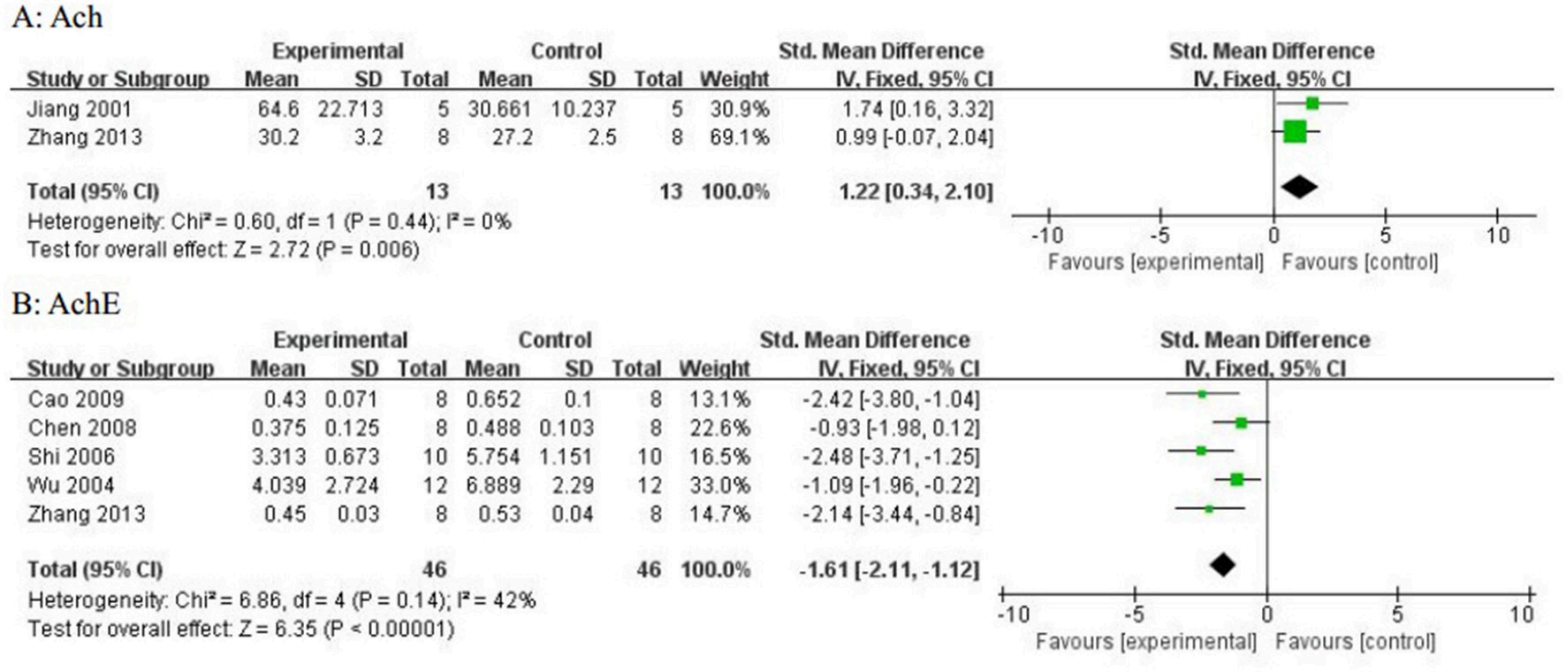
**(A)** The forest plot: effects of textitRhodiola rosea L. for increasing acetylcholine; **(B)** The forest plot:effects of *Rhodiola rosea* L. for decreasing acetylcholinesterase compared with control group.

**Figure 9:**
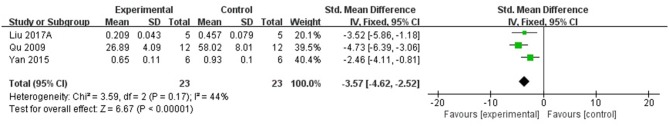
The forest plot: effects of *Rhodiola rosea* L. for decreasing caspase-3 compared with control group.

Compared with controls, meta-analysis of 5 studies (Wang et al., 2008; Qu et al., 2009; Yang et al., 2011a,b; Zhang et al., 2013) showed that *R. rosea* L. significantly increased the level of GSH (*n* = 50, SMD 1.67, 95% CI [1.20 to 2.14], *P* < 0.00001; heterogeneity: χ^2^ = 2.09, df = 4 (*P* = 0.72); *I*^2^ = 0%), (Figure 5); 2 studies (Zhang et al., 2013; Barhwal et al., 2015) for increasing the level of NADH and/or NADPH, (*P* < 0.05); meta-analysis of 12 studies (Shi et al., 2006; Chen, 2008; Zou et al., 2009; Yang et al., 2011a,b; Zhang S. et al., 2012; Zhang X.X. et al., 2012; Zhang et al., 2013; Liu et al., 2017b; Wei, 2017; Xiong and Gao, 2017; Yang, 2017) for increasing SOD level (*n* = 115, SMD 2.12, 95% CI [1.77 to 2.47], P < 0.00001; heterogeneity: χ^2^ = 22.11, df = 11 (*P* = 0.02); *I*^2^ = 50%), (Figure 6); meta-analysis of 12 studies (Shi et al., 2006; Chen, 2008; Qu et al., 2009; Zou et al., 2009; Yang et al., 2011a,b; Zhang X.X. et al., 2012; Zhang et al., 2013; Liu et al., 2017b; Wei, 2017; Xiong and Gao, 2017; Yang, 2017) for reducing MDA level (*n* = 117, SMD−1.89, 95% CI [−2.22 to −1.56], *P* < 0.00001; heterogeneity: χ^2^ = 18.08, df = 11 (*P* = 0.08); *I*^2^ = 39%), (Figure 7); 3 studies (Deng, 2006; Chen, 2008; Wang et al., 2013) for enhancing the expression of NO and/or NOS (*P* < 0.05); meta-analysis of 2 studies (Jiang et al., 2001; Zhang et al., 2013) increasing the activity of Ach (*n* = 13, SMD 1.22, 95% CI [0.34 to 2.10], *P* < 0.00001; heterogeneity: χ^2^ = 0.6, df = 1 (*P* = 0.44); *I*^2^ = 0%), (Figure 8A); meta-analysis of 5 studies (Wu et al., 2004; Shi et al., 2006; Chen, 2008; Cao, 2009; Zhang et al., 2013) down-regulating the activity of AchE (*n* = 46, SMD −1.61, 95% CI [−2.11 to −1.12], P < 0.00001; heterogeneity: χ^2^ = 6.86, df = 4 (*P* = 0.14); *I*^2^ = 42%), (Figure 8B); 3 studies (Chen, 2008; Qu et al., 2009; Qi et al., 2013) for reducing the amount of calcium in nerve cells, (*P* < 0.05); meta-analysis of 3 studies (Qu et al., 2009; Yan et al., 2015; Liu et al., 2017b) for down-regulating the expression of caspase-3 (*n* = 23, SMD −3.57, 95% CI [−4.62 to −2.52], *P* < 0.00001; heterogeneity: χ^2^ = 3.59, df = 2 (*P* = 0.17); *I*^2^ = 44%), (Figure 9); 5 studies (Cao, 2009; Yan et al., 2015; Guo et al., 2017; Liu et al., 2017a; Wei, 2017) for increasing the expression of Bcl-2 and reducing the expression of Bax protein in the hippocampus,(*P* < 0.05); 1 study (Zou et al., 2009) for inhibiting the expression of TNF-α; 1 study (Zhang et al., 2013) for inhibiting the expression of nuclear factor κB (NF-κB).

### Subgroup Analysis and Sensitivity Analysis

**Figure 10:**
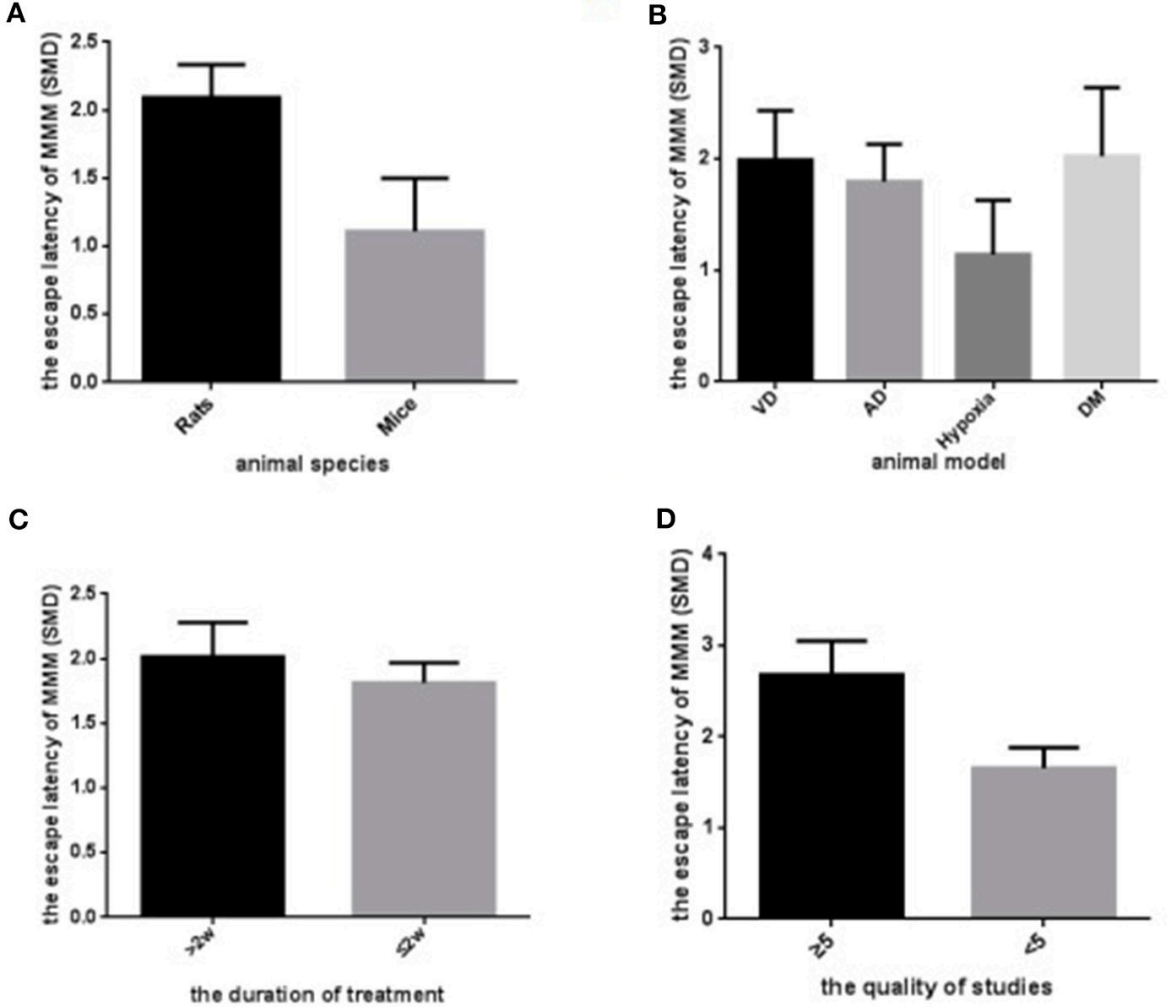
Subgroup analyses of the escape latency. **(A)** The animal species on the effect size of the outcome measure; **(B)** the animal model on the effect size of the outcome measure; **(C)** the duration of the treatment on the effect size of the outcome measure; **(D)** the quality of studies on the effect size of the outcome measure. The magnitude of absolute value SMD reflected the effect size.

To explore potential confounding factors which affected the outcome measures, we stratified analysis of the escape latency based on variables including animal species, animal model, the duration of treatment, and the quality of study. In the subgroup analysis of these factors, the effect size of rat species was larger than mice (SMD = −2.09 vs. SMD = −1.08, Figure 10A). Animal model showed great discrepancy in the overall effect of outcome measure, which the model of hypobaric hypoxia with scale of 16.4% weight accounted for smaller effect size than any other model (SMD = −1.18 vs. SMD _pooled_ = −1.96, Figure 10B). The longer period of *R. rosea* L. treatment also showed greater effect size than the shorter treatment with 2 weeks or less (SMD = −1.92 vs. SMD = −1.83, Figure 10C). Notably, the lower quality studies did not exhibit larger effect size than the higher ones (SMD = −1.65 vs. SMD = −2.55, Figure 10D).

Sensitivity analyses showed that the results did not substantially alter after removing any one trial. However, when we only include studies using mice as animal models, meta-analysis of 5 studies (Liu et al., 2003; Wu et al., 2004; Deng, 2006; Wang et al., 2013; Ge et al., 2017) showed a small difference in favor of *R. rosea* L. treatment compared with control groups with lower heterogeneity (*n* = 61, SMD = −1.08, 95%CI [−1.47, −0.68], *P* < 0.00001; Heterogeneity: χ^2^ = 6.22, df = 4 (*P* < 0.00001); *I*^2^ = 36%).

## Discussion

### Summary of Evidence

In this meta-analysis, we assessed *R. rosea* L. treatment on learning and memory function based on 36 eligible studies. The results revealed that *R. rosea* L. could evidently reduce the escape latency, improve the frequency and the length of time spent in MWM and decrease the number of errors in step down test, dark avoidance test, and Y maze when comparing with control groups in animal models.

### Limitations

**Figure 11:**
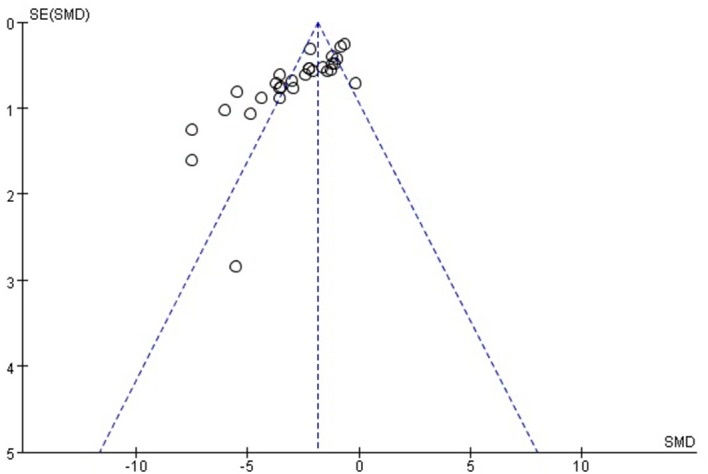
The funnel plot: effects of *Rhodiola rosea* L. for decreasing the escape latency in MWM.

Some limitations should be considered while interpreting this study. First, the methodological quality of the included studies was considerably variable and inferior. Nearly all of the included studies had an overall assessment as “high risk of bias,” so we could not exclude that our results may be biased. Second, calculation of sample size and blindness of model establishment and outcome measurement are pivotal in quality control of research, yet no studies provided these critical information in this systematic review. Third, it's not worthy that almost all the included studies declared random allocation to treatment and control groups, while the detailed procedure was not supported at all. Additionally, gender difference was overlooked in the included study. Male/female mice models were used in the two studies (Wu et al., 2004; Wang et al., 2013) for cognitive experiments. Although the mechanism is unclear, a male advantage for working memory and a female advantage for visual memory and social cognition in rodent models were highlighted (Leger and Neill, 2016). Moreover, funnel plots (Figure 11) showed potential publication bias existed in this research field, suggesting studies with null effect are missing. Studies achieved statistically significant outcomes have been shown to be three times more likely to be published than that with null outcomes (Dickersin et al., 1987). Publication bias is due to multiple factor such as researchers and journal editors prefer positive results rather than negative or inconclusive results (Wolfgang, 2007). Thus, the effect of *R. rosea* L. on learning and memory function cannot be excluded from overall over estimation of effect sizes and efficacy, which may weaken the validity of conclusions.

### Interpretation of the Results

Considerably high heterogeneity was present in this meta-analysis, the summary positive results should be interpreted with caution. Given that there are many potential sources of heterogeneity in the outcome, several means are taken into consideration for the finding of the causes. Firstly, random-effects models are used in our study. Heterogeneity is a key condition for the execution of meta-regression, but it can also cause confusion if confounding factors are not well-balanced. As small number of studies were included in this meta-analysis, we made the meta-regression with reservations and did subgroup analysis based on four potential confounding factors including animal species, animal model, the duration of treatment, and the quality of study. The results of subgroup analyses suggested that the first three factors were very likely to be the sources of heterogeneity in this research, while the poor quality of methodology still could not be exempted from the excuses for high heterogeneity. Sensitivity analyses have also been adopted to detect the effects of studies identified as being aberrant result, or being highly influential in the analysis (Haidich, 2010). While no studies identified as being aberrant result or being highly influential in the analysis from the results of sensitivity analyses in this review.

### Implication for Further Studies

While mice models are increasing used for cognitive experiments involving learning and memory process that were originally designed for rat species, the stability of spatial cognitive representation in rats changes more slightly over time than in mice (Hok et al., 2016). In the subgroup analyses, rat species also showed greater effect size in depression of escape latency than that of mice. Thus, rat species were considered as suitable cognitive experiments involving learning and memory process. In addition, the impact of gender on cognitive function deserves attention. In the present study, male rats models and male/female mice models were used in the included studies of our review for working memory process, while no significant difference existed in the pooled result of meta-analysis in escape latency of MWM test after discarding two studies with male/female mice (Wu et al., 2004; Wang et al., 2013). However, a male advantage for working memory and a female advantage for visual memory and social cognition in rodent models were highlighted in recent systematic review (Leger and Neill, 2016). Thus, using a single sex animal model is considered more reasonable for study learning and memory function in future experiments.

Two dementia models of AD and vascular dementia (VaD) are most commonly approached for learning and memory research (Kalaria et al., 2008). However, there are several model methods for inducing these two dementia types and their differences of effectiveness and robustness are not investigated. For this systematic review, intra-peritoneal injection with scopolamine, combination with aluminum trichloride, D-galactose and scopolamine, intracerebroventricular injection with streptozotocin, and hippocampal injection with Aβ_1−40_ were the most approaches for AD models in the included studies. Different time scales of artery occlusion and different arteries selected for blood blocking were adopted for VaD models. In the subgroup analyses, six animal models including AD, VaD, hypoxia, sleep deprivation, epilepsy, and diabetes mellitus models were conducted for cognitive impairment, of which AD models accounted for 38.7% weight and VaD models accounted for 17.7% weight. These two most weight of models showed no significant difference in effect size on escape latency of MWM test, which can indirectly reflect the effectiveness and robustness of the two dementia models for cognitive impairment.

A lower-quality study trends toward better outcomes, leading to the global estimated effect overstated (García-Bonilla et al., 2012). In the present study, many domains had flaws in aspects of randomization, allocation concealment, and blinding and sample size calculation, which are the core standards of study design (Moher et al., 2015). Thus, we recommended that the experimental research of *R. rosea* L. for learning and memory function need be promoted by means of incorporating the ARRIVE guidelines (Kilkenny et al., 2012).

Long-term treatment for dementia progression with Gingko biloba showed great effect on prevention of cognitive decline (Dodge et al., 2008). In parallel, treatment with *R. rosea* L. more than 2 weeks showed greater effect size in the escape latency of MWM test than that of < 2 weeks' treatment in the subgroup analyses, suggesting that long-term treatment with *R. rosea* L. has a greater benefit for cognitive function. In view of the number of studies in subgroup analyses was relatively small and may lack of statistical power to detect smaller effect sizes. Therefore, we recommend that future studies involving this problem are conducted strictly complying with standards of research methodology and report their adequate information clearly.

**Figure 12:**
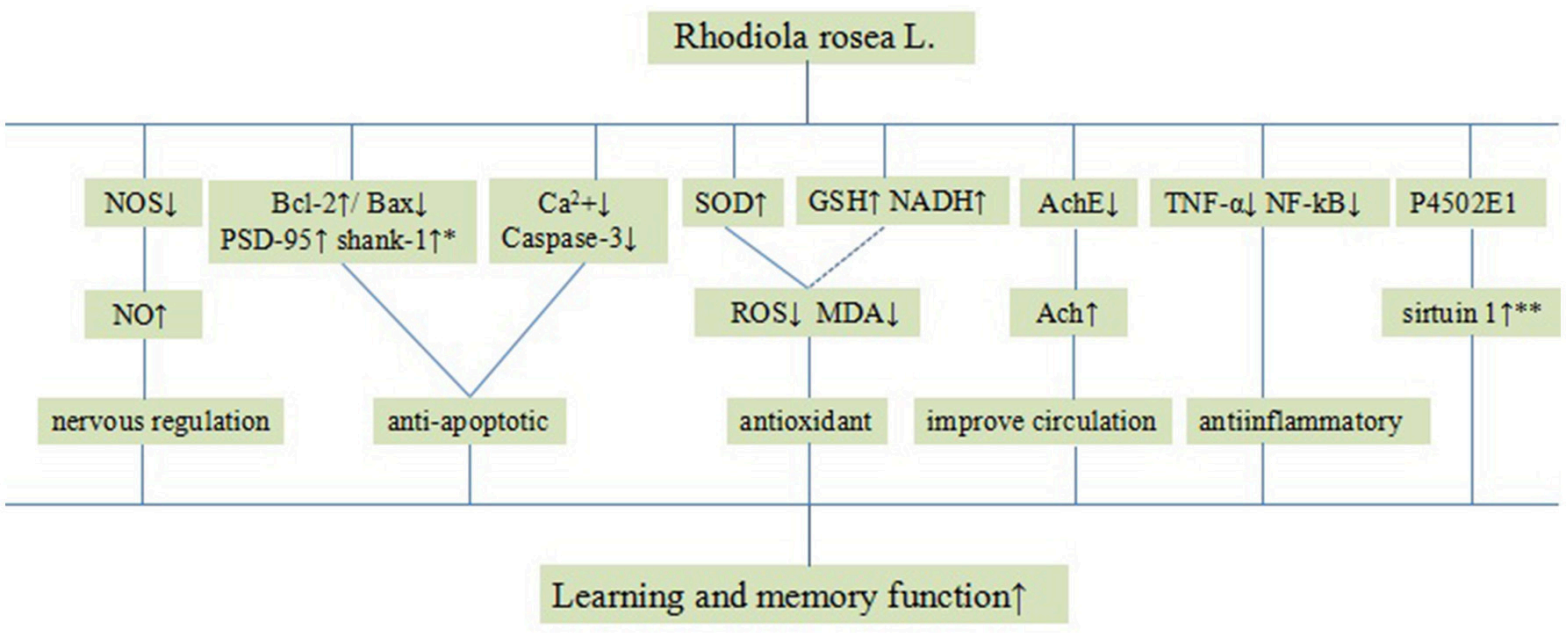
A schematic representation of possible mechanisms of *Rhodiola rosea* L. for improving learning and memory function. Solid lines indicate established effects, whereas dashed lines represent putative mechanisms. *The expression of Bcl-2, Bax protein, PSD-95 and shank-1 protein in the hippocampus; **The activity of sirtuin 1.

Systemic review of animal studies plays a critical role in drug development and the clarification of physiological and pathological mechanisms of clinical research. In this systematic review, some included studies speculated on how *R. rosea* L. enhanced learning and memory function and the possible mechanisms are summarized as follows: (1) antioxidant through increasing the level of GSH (Wang et al., 2008; Qu et al., 2009; Yang et al., 2011a,b; Zhang et al., 2013), NADH/NADPH (Zhang et al., 2013; Barhwal et al., 2015), and enhancing SOD-induced antioxidant via attenuating chondriokinesis to reduce the release of MDA (Jiang et al., 2001; Shi et al., 2006; Chen, 2008; Qu et al., 2009; Zou et al., 2009; Yang et al., 2011a,b; Zhang S. et al., 2012; Zhang X.X. et al., 2012; Zhang et al., 2013; Liu et al., 2017b; Wei, 2017; Xiong and Gao, 2017; Yang, 2017); (2) improvement of the circulation by enhancing the expression of NO via up-regulating the expression of NOS (Deng, 2006; Chen, 2008; Wang et al., 2013); (3) cholinergic regulation through increasing the activity of Ach via down-regulating the activity of AchE (Jiang et al., 2001; Xie, 2003; Wu et al., 2004; Shi et al., 2006; Chen, 2008; Cao, 2009; Zhang et al., 2013); (4) inhibition of apoptosis through reducing the amount of calcium in nerve cells (Chen, 2008; Qu et al., 2009; Qi et al., 2013) and down-regulating the expression of caspase-3 (Qu et al., 2009; Yan et al., 2015; Liu et al., 2017b); (5) anti-inflammatory through inhibiting the expression of TNF-α (Zou et al., 2009) and NF-κB (Zhang et al., 2013); (6) increasing sirtuin 1 (SIRT1) activity through a cytochrome P4502E1 (CYP2E1)-regulated mechanism (Cao, 2009); (7) increasing the expression of Bcl-2 and reducing the expression of Bax protein in the hippocampus (Cao, 2009; Yan et al., 2015; Guo et al., 2017; Liu et al., 2017a; Wei, 2017) and improving the expression of PSD-95 and shank-1 protein in the hippocampus (Wang et al., 2008), alleviating apoptosis in the hippocampal CA1 area. The possible mechanisms of *R. rosea* L. for learning and/or memory function are through antioxidant, cholinergic regulation, anti-apoptosis activities, anti-inflammatory, improving coronary blood flow, and cerebral metabolism (Figure 12).

## Conclusion

We have provided a first-ever comprehensive preclinical systematic review of *R. rosea* L. for cognitive behavior in animal studies and our findings indicate that *R. rosea* L. improves learning and memory function in experimental models.

## Author Contributions

GM, QZ, MX, XZ, ZL, LL, and GZ designed the study. GM and QZ collected the data. GM and MX performed all analyses. GM, QZ, ZL, LL, and GZ wrote the manuscript. All authors contributed to writing of this manuscript.

### Conflict of Interest Statement

The authors declare that the research was conducted in the absence of any commercial or financial relationships that could be construed as a potential conflict of interest.
